# 1-(3-Cyano­phen­yl)-3-(2-furo­yl)thio­urea

**DOI:** 10.1107/S1600536808016012

**Published:** 2008-06-07

**Authors:** Jahyr E. Theodoro, Yvonne Mascarenhas, Javier Ellena, Osvaldo Estévez-Hernández, Julio Duque

**Affiliations:** a Grupo de Cristalografía, Instituto de Física de São Carlos, Universidade de São Paulo, São Carlos, Brazil; b Instituto de Física de São Carlos, Universidade de São Paulo, São Carlos, Brazil; c Department of Structure Analysis, Institute of Materials, University of Havana, Cuba

## Abstract

The title compound, C_13_H_9_N_3_O_2_S, was synthesized from furoyl isothio­cyanate and 3-amino­benzonitrile in dry acetone. The thio­urea group is in the thio­amide form. The thio­urea fragment makes dihedral angles of 3.91 (16) and 37.83 (12)° with the ketofuran group and the benzene ring, respectively. The mol­ecular geometry is stabilized by N—H⋯O hydrogen bonds. In the crystal structure, centrosymmetrically related mol­ecules are linked by two inter­molecular N—H⋯S hydrogen bonds to form dimers.

## Related literature

For general background, see: Aly *et al.* (2007 ▶); Koch (2001 ▶). For related structures, see: Dago *et al.* (1987 ▶); Otazo-Sánchez *et al.* (2001 ▶); Pérez *et al.* (2008 ▶); Duque *et al.* (2008 ▶). For the synthesis, see: Otazo-Sánchez *et al.* (2001 ▶).
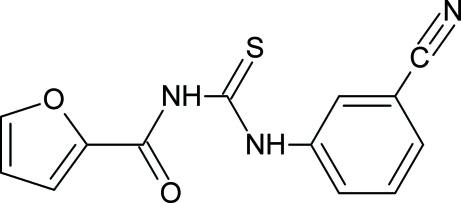


## Experimental

### 

#### Crystal data

C_13_H_9_N_3_O_2_S
*M* *_r_* = 271.29
Monoclinic, 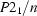

*a* = 16.7375 (5) Å
*b* = 3.8789 (1) Å
*c* = 19.6739 (5) Å
β = 96.956 (1)°
*V* = 1267.89 (6) Å^3^
*Z* = 4
Mo *K*α radiation
μ = 0.26 mm^−1^
*T* = 294 K
0.16 × 0.04 × 0.03 mm

#### Data collection

Nonius KappaCCD diffractometer
Absorption correction: none
4807 measured reflections
2684 independent reflections
1908 reflections with *I* > 2σ(*I*)
*R*_int_ = 0.040

#### Refinement

*R*[*F* ^2^ > 2σ(*F* ^2^)] = 0.070
*wR*(*F* ^2^) = 0.208
*S* = 1.08
2684 reflections
172 parameters
H-atom parameters constrained
Δρ_max_ = 0.51 e Å^−3^
Δρ_min_ = −0.34 e Å^−3^

### 

Data collection: *COLLECT* (Enraf–Nonius, 2000 ▶); cell refinement: *SCALEPACK* (Otwinowski & Minor, 1997 ▶); data reduction: *DENZO* (Otwinowski & Minor, 1997 ▶) and *SCALEPACK*; program(s) used to solve structure: *SHELXS97* (Sheldrick, 2008 ▶); program(s) used to refine structure: *SHELXL97* (Sheldrick, 2008 ▶); molecular graphics: *ORTEP-3 for Windows* (Farrugia, 1997 ▶); software used to prepare material for publication: *WinGX* (Farrugia, 1999 ▶).

## Supplementary Material

Crystal structure: contains datablocks global, I. DOI: 10.1107/S1600536808016012/rz2219sup1.cif

Structure factors: contains datablocks I. DOI: 10.1107/S1600536808016012/rz2219Isup2.hkl

Additional supplementary materials:  crystallographic information; 3D view; checkCIF report

## Figures and Tables

**Table 1:** Hydrogen-bond geometry (Å, °)

*D*—H⋯*A*	*D*—H	H⋯*A*	*D*⋯*A*	*D*—H⋯*A*
N1—H1⋯O2	0.86	2.28	2.701 (5)	110
N1—H1⋯S1^i^	0.86	2.80	3.629 (4)	163
N2—H2⋯O1	0.86	1.90	2.622 (4)	141

Symmetry code: (i) 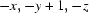.

**Fig. 1.**
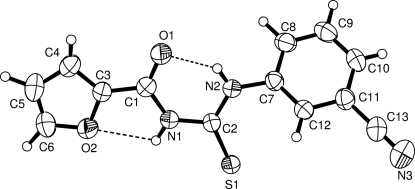
View of the molecule (50% probability displacement ellipsoids) Intramolecular hydrogen bonds are shown as dashed lines.

**Fig. 2.**
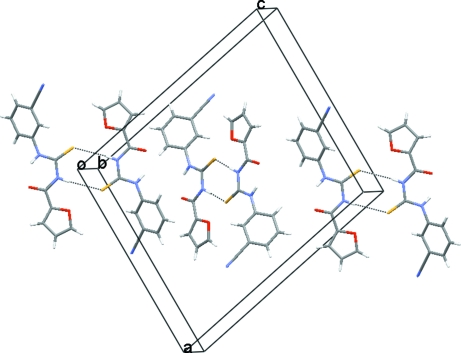
View of the crystal packing of the title compound. Intermolecular hydrogen bonds are shown as dashed lines.

## supplementary crystallographic information

### Comment

The importance of aroylthioureas is found largely in heterocyclic syntheses and many of these substrates have interesting biological activities. Aroylthioureas have also been found to have applications in metal complexes and molecular electronics (Aly *et al.*, 2007). Structural determinations of this kind of derivatives shed more light on the chemistry of aroylthiourea compounds and their wide variety of applications. The title compound (Fig. 1) is another example of our newly synthesized furoylthiourea derivatives.

The title compound crystallizes in the thioamide form. The bond lengths are within the ranges observed for similar compounds (Koch, 2001). The C2—S1 and C1—O1 bonds (Table 1) both show the expected double-bond character. The short values of the C2—N1 (1.347 (5) Å), C2—N2 (1.348 (5) Å) and C1—N1 (1.368 (5) Å) bonds indicate partial double bond character. These results can be explained by the existence of resonance in this part of the molecule. The furan carbonyl group is nearly coplanar with the plane of the thiourea fragment (dihedral angle 3.39(16°), whereas the benzene ring is inclined by 37.83 (12)°. The geometry in the thiourea group is stabilized by the N2—H2···O1 and N1—H1···O2 intramolecular hydrogen bonds (Fig. 1 and Table 2). The crystal structure is stabilized by two intermolecular N1—H1···S1 hydrogen bonds (Fig. 2 and Table 2) between centrosymmetrically related molecules forming dimers stacked along the [010] direction.

### Experimental

The title compound was synthesized according to a previous report (Otazo-Sánchez *et al.*, 2001), by converting furoyl chloride into furoyl isothiocyanate and then condensing with 3-aminobenzonitrile. The resulting solid product was crystallized from ethanol yielding X-ray quality single crystals (m.p 148–149 °C). Elemental analysis (%) for C_13_H_9_N_3_O_2_S calculated: C 57.56, H 3.32, N 15.50, S 11.81; found: C 57.77, H 3.34, N 15.79, S 11.73.

### Refinement

H atoms were placed in calculated positions with N—H = 0.88 Å and C—H = 0.95 Å or 0.98 Å (methylene), and refined in riding-model, with *U*_iso_(H) = 1.5*U*_eq_(C) for methylene or 1.2*U*_eq_(C,N) for others.

### Crystal data

**Table:**

C_13_H_9_N_3_O_2_S	*F*_000_ = 560
*M**_r_* = 271.29	*D*_x_ = 1.421 Mg m^−^^3^
Monoclinic, *P*2_1_/*n*	Mo *K*α radiation λ = 0.71073 Å
Hall symbol: -P 2yn	Cell parameters from 3051 reflections
*a* = 16.7375 (5) Å	θ = 2.9–26.7º
*b* = 3.87890 (10) Å	µ = 0.26 mm^−^^1^
*c* = 19.6739 (5) Å	*T* = 294 K
β = 96.9560 (10)º	Prism, colourless
*V* = 1267.89 (6) Å^3^	0.16 × 0.04 × 0.03 mm
*Z* = 4	

### Data collection

**Table:**

Enraf–Nonius KappaCCD diffractometer	*R*_int_ = 0.040
CCD rotation images, thick slices scans	θ_max_ = 26.9º
Absorption correction: none	θ_min_ = 3.9º
4807 measured reflections	*h* = −20→21
2684 independent reflections	*k* = −4→4
1908 reflections with *I* > 2σ(*I*)	*l* = −25→24

### Refinement

**Table:**

Refinement on *F*^2^	H-atom parameters constrained
Least-squares matrix: full	*w* = 1/[σ^2^(*F*_o_^2^) + (0.0641*P*)^2^ + 3.4802*P*] where *P* = (*F*_o_^2^ + 2*F*_c_^2^)/3
*R*[*F*^2^ > 2σ(*F*^2^)] = 0.071	(Δ/σ)_max_ < 0.001
*wR*(*F*^2^) = 0.208	Δρ_max_ = 0.51 e Å^−^^3^
*S* = 1.08	Δρ_min_ = −0.34 e Å^−^^3^
2684 reflections	Extinction correction: none
172 parameters	

### Special details

**Table:**

Geometry. All e.s.d.'s (except the e.s.d. in the dihedral angle between two l.s. planes) are estimated using the full covariance matrix. The cell e.s.d.'s are taken into account individually in the estimation of e.s.d.'s in distances, angles and torsion angles; correlations between e.s.d.'s in cell parameters are only used when they are defined by crystal symmetry. An approximate (isotropic) treatment of cell e.s.d.'s is used for estimating e.s.d.'s involving l.s. planes.

### Fractional atomic coordinates and isotropic or equivalent isotropic displacement parameters (Å^2^)

**Table:**

	*x*	*y*	*z*	*U*_iso_*/*U*_eq_	
S1	0.10993 (6)	0.7197 (3)	−0.00840 (5)	0.0465 (3)	
O1	0.08926 (19)	0.9883 (11)	0.21366 (15)	0.0699 (11)	
C13	0.3735 (3)	1.3099 (15)	−0.0369 (3)	0.0617 (13)	
N3	0.3872 (3)	1.4227 (17)	−0.0884 (3)	0.0909 (17)	
N1	0.0452 (2)	0.7949 (10)	0.10538 (16)	0.0457 (9)	
H1	0.0031	0.7186	0.0805	0.055*	
O2	−0.10121 (19)	0.6665 (10)	0.14431 (15)	0.0643 (10)	
N2	0.17909 (19)	0.9396 (11)	0.11374 (17)	0.0482 (9)	
H2	0.1712	0.991	0.1549	0.058*	
C7	0.2586 (2)	0.9846 (11)	0.0976 (2)	0.0415 (9)	
C5	−0.1517 (3)	0.7102 (15)	0.2424 (3)	0.0676 (15)	
H5	−0.1872	0.6986	0.2752	0.081*	
C2	0.1143 (2)	0.8261 (11)	0.07249 (19)	0.0398 (9)	
C9	0.3556 (2)	1.1694 (12)	0.0263 (2)	0.0474 (10)	
C8	0.2756 (2)	1.1209 (12)	0.0366 (2)	0.0456 (10)	
H8	0.2342	1.1798	0.0026	0.055*	
C3	−0.0430 (2)	0.7960 (12)	0.1925 (2)	0.0471 (10)	
C10	0.4180 (3)	1.0819 (14)	0.0767 (2)	0.0571 (12)	
H10	0.4714	1.1093	0.069	0.068*	
C1	0.0355 (3)	0.8694 (13)	0.1719 (2)	0.0495 (11)	
C11	0.3993 (3)	0.9541 (15)	0.1381 (3)	0.0633 (14)	
H11	0.4403	0.9026	0.1729	0.076*	
C4	−0.0724 (3)	0.8274 (14)	0.2531 (2)	0.0581 (13)	
H4	−0.0452	0.9105	0.2938	0.07*	
C6	−0.1676 (3)	0.6183 (17)	0.1772 (3)	0.0705 (15)	
H6	−0.2168	0.5335	0.1569	0.085*	
C12	0.3204 (3)	0.9022 (13)	0.1483 (2)	0.0535 (11)	
H12	0.3084	0.8109	0.1895	0.064*	

### Atomic displacement parameters (Å^2^)

**Table:**

	*U*^11^	*U*^22^	*U*^33^	*U*^12^	*U*^13^	*U*^23^
S1	0.0410 (6)	0.0603 (8)	0.0382 (5)	−0.0046 (5)	0.0050 (4)	−0.0056 (5)
O1	0.0528 (19)	0.112 (3)	0.0457 (17)	−0.021 (2)	0.0093 (14)	−0.0222 (19)
C13	0.049 (3)	0.074 (4)	0.063 (3)	−0.004 (2)	0.015 (2)	0.004 (3)
N3	0.088 (4)	0.115 (5)	0.073 (3)	−0.013 (3)	0.025 (3)	0.017 (3)
N1	0.0389 (17)	0.061 (2)	0.0367 (17)	−0.0055 (17)	0.0040 (14)	−0.0029 (17)
O2	0.0536 (18)	0.096 (3)	0.0440 (16)	−0.0177 (19)	0.0072 (14)	−0.0081 (18)
N2	0.0393 (18)	0.069 (3)	0.0355 (17)	−0.0083 (18)	0.0028 (13)	−0.0001 (17)
C7	0.039 (2)	0.041 (2)	0.045 (2)	−0.0045 (18)	0.0045 (16)	0.0001 (18)
C5	0.058 (3)	0.075 (4)	0.075 (3)	0.001 (3)	0.031 (3)	−0.004 (3)
C2	0.0358 (19)	0.043 (2)	0.040 (2)	−0.0019 (17)	0.0049 (16)	0.0019 (18)
C9	0.044 (2)	0.046 (3)	0.053 (2)	−0.0032 (19)	0.0071 (18)	0.003 (2)
C8	0.039 (2)	0.052 (3)	0.044 (2)	−0.0024 (19)	0.0022 (17)	0.0050 (19)
C3	0.042 (2)	0.058 (3)	0.042 (2)	−0.004 (2)	0.0073 (17)	−0.003 (2)
C10	0.036 (2)	0.071 (3)	0.064 (3)	−0.005 (2)	0.005 (2)	0.002 (3)
C1	0.047 (2)	0.060 (3)	0.042 (2)	−0.005 (2)	0.0072 (18)	−0.001 (2)
C11	0.043 (2)	0.081 (4)	0.062 (3)	−0.001 (2)	−0.011 (2)	0.010 (3)
C4	0.059 (3)	0.073 (4)	0.045 (2)	−0.003 (3)	0.016 (2)	−0.009 (2)
C6	0.045 (3)	0.094 (4)	0.073 (3)	−0.014 (3)	0.013 (2)	0.009 (3)
C12	0.051 (2)	0.060 (3)	0.047 (2)	−0.008 (2)	−0.0041 (19)	0.006 (2)

### Geometric parameters (Å, °)

**Table:**

S1—C2	1.637 (4)	C5—C4	1.395 (7)
O1—C1	1.233 (5)	C5—H5	0.93
C13—N3	1.153 (6)	C9—C8	1.392 (6)
C13—C9	1.422 (6)	C9—C10	1.392 (6)
N1—C1	1.368 (5)	C8—H8	0.93
N1—C2	1.397 (5)	C3—C4	1.348 (6)
N1—H1	0.86	C3—C1	1.450 (6)
O2—C6	1.365 (6)	C10—C11	1.377 (7)
O2—C3	1.369 (5)	C10—H10	0.93
N2—C2	1.348 (5)	C11—C12	1.375 (6)
N2—C7	1.416 (5)	C11—H11	0.93
N2—H2	0.86	C4—H4	0.93
C7—C8	1.373 (6)	C6—H6	0.93
C7—C12	1.383 (6)	C12—H12	0.93
C5—C6	1.326 (7)		
			
N3—C13—C9	179.3 (6)	C9—C8—H8	120.5
C1—N1—C2	128.7 (3)	C4—C3—O2	109.9 (4)
C1—N1—H1	115.6	C4—C3—C1	132.0 (4)
C2—N1—H1	115.6	O2—C3—C1	118.1 (4)
C6—O2—C3	105.9 (4)	C11—C10—C9	118.9 (4)
C2—N2—C7	128.0 (3)	C11—C10—H10	120.6
C2—N2—H2	116	C9—C10—H10	120.6
C7—N2—H2	116	O1—C1—N1	123.7 (4)
C8—C7—C12	120.2 (4)	O1—C1—C3	120.0 (4)
C8—C7—N2	122.9 (4)	N1—C1—C3	116.3 (4)
C12—C7—N2	116.8 (4)	C12—C11—C10	120.3 (4)
C6—C5—C4	108.0 (4)	C12—C11—H11	119.9
C6—C5—H5	126	C10—C11—H11	119.9
C4—C5—H5	126	C3—C4—C5	106.3 (4)
N2—C2—N1	113.6 (3)	C3—C4—H4	126.9
N2—C2—S1	127.3 (3)	C5—C4—H4	126.9
N1—C2—S1	119.1 (3)	C5—C6—O2	110.0 (4)
C8—C9—C10	121.1 (4)	C5—C6—H6	125
C8—C9—C13	119.1 (4)	O2—C6—H6	125
C10—C9—C13	119.8 (4)	C11—C12—C7	120.6 (4)
C7—C8—C9	118.9 (4)	C11—C12—H12	119.7
C7—C8—H8	120.5	C7—C12—H12	119.7
			
C2—N2—C7—C8	41.1 (7)	C2—N1—C1—C3	−177.3 (4)
C2—N2—C7—C12	−142.4 (5)	C4—C3—C1—O1	−1.2 (9)
C7—N2—C2—N1	176.9 (4)	O2—C3—C1—O1	179.5 (5)
C7—N2—C2—S1	−1.9 (7)	C4—C3—C1—N1	177.9 (5)
C1—N1—C2—N2	1.3 (7)	O2—C3—C1—N1	−1.3 (7)
C1—N1—C2—S1	−179.8 (4)	C9—C10—C11—C12	2.4 (8)
C12—C7—C8—C9	0.9 (7)	O2—C3—C4—C5	0.5 (6)
N2—C7—C8—C9	177.3 (4)	C1—C3—C4—C5	−178.8 (5)
C10—C9—C8—C7	0.1 (7)	C6—C5—C4—C3	−0.7 (7)
C13—C9—C8—C7	179.9 (5)	C4—C5—C6—O2	0.7 (7)
C6—O2—C3—C4	−0.1 (6)	C3—O2—C6—C5	−0.4 (6)
C6—O2—C3—C1	179.3 (5)	C10—C11—C12—C7	−1.5 (8)
C8—C9—C10—C11	−1.7 (8)	C8—C7—C12—C11	−0.3 (7)
C13—C9—C10—C11	178.4 (5)	N2—C7—C12—C11	−176.8 (5)
C2—N1—C1—O1	1.8 (8)		

### Hydrogen-bond geometry (Å, °)

**Table:**

*D*—H···*A*	*D*—H	H···*A*	*D*···*A*	*D*—H···*A*
N1—H1···O2	0.86	2.28	2.701 (5)	110
N1—H1···S1^i^	0.86	2.80	3.629 (4)	163
N2—H2···O1	0.86	1.90	2.622 (4)	141

Symmetry codes: (i) −*x*, −*y*+1, −*z*.
